# Breast Cancer During Pregnancy: A Marked Propensity to Triple-Negative Phenotype

**DOI:** 10.3389/fonc.2020.580345

**Published:** 2020-12-23

**Authors:** Soumaya Allouch, Ishita Gupta, Shaza Malik, Halema F. Al Farsi, Semir Vranic, Ala-Eddin Al Moustafa

**Affiliations:** ^1^College of Medicine, QU Health, Qatar University, Doha, Qatar; ^2^Biomedical Research Center, Qatar University, Doha, Qatar

**Keywords:** breast cancer, pregnancy, gene deregulation, delayed childbearing, triple-negative

## Abstract

Breast and cervical cancers comprise 50% of all cancers during pregnancy. In particular, gestational breast cancer is considered one of the most aggressive types of cancers, which is a rare but fatal disease. However, the incidence of this type of cancer is increasing over the years and its prevalence is expected to rise further as more women delay childbearing. Breast cancer occurring after pregnancy is generally triple negative with specific characterizations of a poorer prognosis and outcome. On the other hand, it has been pointed out that this cancer is associated with a specific group of genes which can be used as precise targets to manage this deadly disease. Indeed, combination therapies consisting of gene-based agents with other cancer therapeutics is presently under consideration. We herein review recent progress in understanding the development of breast cancer during pregnancy and their unique subtype of triple negative which is the hallmark of this type of breast cancer.

## Introduction

Breast cancer is the most common type of cancer in females affecting more than 2.1 million women and causing more than half a million deaths annually (1). The etiology of breast cancer is complex and heterogeneous with numerous pathological characteristics; these directly correlate with available treatment options and disease prognosis (2). Based on microarray and unsupervised cluster analysis studies, breast cancer is classified into four molecular subtypes with distinct gene expression patterns and clinical outcomes (3). These subtypes include luminal (A and B), human epidermal growth factor receptor 2 (HER2)-type, and triple-negative breast cancers (TNBC) (4–6).

TNBC possesses molecular characteristics and clinical aggressiveness that is analogous to that of basal-like cancer (7). TNBC lacks estrogen receptor (ER), progesterone receptor (PR), and HER2 expression and accounts for ~15% of all breast cancer cases (7). More intriguingly, the described subtype of breast cancer is reportedly associated with high-grade invasive ductal carcinomas and, when compared with other subtypes, TNBC was found to be larger with higher metastatic propensity to lungs, brain and other visceral organs.

Since the majority of basal-like cancers are also TNBC and more than 80% of TNBC are basal-like breast cancers, it has been postulated that TNBC and basal-like phenotypes are essentially analogous (8). Using gene expression profiling, the molecular heterogeneity of TNBC was well defined. One study subclassified TNBC into six molecular subtypes including basal-like 1, basal-like 2, immunomodulatory, mesenchymal-like, mesenchymal stem-like, and luminal androgen receptor (LAR) subtype (9). Furthermore, TNBC molecular subtyping revealed three subtypes, LAR, basal-like with low immune response and high M2-like macrophages and basal-enriched with high immune response and low M2-like macrophages (10). Despite histological differences, the metastatic characteristics of the highlighted TNBC subtypes remain comparable (11). In addition to the metastatic potential of TNBC, it is vital to note that once TNBC metastasizes the window between relapse and death becomes very narrow (12). Dent et al. reports that patients with TNBC were more likely to experience significant relapses and higher rates of death when compared with women suffering from other types of breast cancers (12). The same group also reports a four folds increase in the likelihood of visceral metastasis in TNBC patients when compared with other types of breast cancer (13).

Breast cancer risk factors are various; nonetheless, a strong association between pregnancy and breast cancer has been well established (14, 15). Although early age pregnancy is considered generally protective against breast cancer, this protection is deferred. Nevertheless, the period immediately subsequent to pregnancy is characterized by a risk of breast cancer development (16). During the last 30 years, diagnosis of cancer during pregnancy has become more common due to the present trend of delaying pregnancy or childbearing to an older age (17). Pregnancy-associated breast cancer (PABC) is an upcoming issue; in this review, we aim at illustrating recent advances in understanding the development and progression of PABC and their associated genes with emphasis on TNBC to review current and potential management options.

Gestational cancer is defined as cancer diagnosed during pregnancy or the first postpartum year (18). Pregnancy-associated melanoma, breast and cervical cancers are the most common malignancies during pregnancy; both cervical and breast cancers account for 50% of all gestational cancers (19). Hematological cancers including leukemia and lymphoma comprise 25% of gestational cancer cases, while ovarian, thyroid and colon cancers are less common (19).

Pregnancy-associated breast cancer (PABC), also known as “gestational breast cancer” is defined as breast cancer diagnosed either during pregnancy or up to one year postnatal (20) and affects around 1 in 3,000 pregnant women (21). In comparison with nulliparous women, breast cancer in pregnant women is histologically similar; approximately 75%–90% of the tumors are invasive ductal carcinomas with no-special-type (NST) (21–26). While, invasive lobular carcinoma (ILC) and other histological types are uncommon in patients with PABC (23, 27–29). Previous studies have showed that postpartum period is linked to a higher risk of developing more aggressive, high-grade breast cancer (14, 16, 23, 26, 30–32) with high tumor nuclear grade (29, 33, 34) and poorly differentiated tumors (24). PABC is also associated with lymphovascular invasion (22, 23, 33), more frequent lymph node involvement and larger tumor size (21–23, 27, 35–40). Similar to nulliparous women, PABC tends to commonly metastasize to lung, liver, brain, and skeletal system (41). Women with PABC have a poorer clinical outcome and disease-free survival with a higher mortality rate compared with nulliparous women (42–45).

With regards to steroid receptors, the previous data showed that estrogen and progesterone play major roles in breast tumorigenesis (46–48), and their effects on breast cells are mediated by their respective receptors, the ER and the PR (49, 50). Earlier studies evaluated tumor histology as well as the prognostic and predictive markers (ER, PR, HER‐2/*neu*, p53, and Ki‐67) in PABC; in comparison to age-matched non-pregnant women, their findings show that PABC exhibit lower expression of ER/PR and higher expression Ki‐67, p53 HER2 (23, 25, 26, 43, 51–53). However, a study by Shousha showed that during pregnancy or early lactation, the expression of HER-2/*neu* was negative; however HER2 expression was noticed after delivery or at the end of lactation indicating suppression of HER-2/*neu* expression during pregnancy and lactation (32). Low ER positivity was observed in women with PABC, plausibly due to decreased ER levels during pregnancy (22, 51, 54, 55). It has been indicated that increased estrogen levels can aid in preventing ER-positive tumors (56). Furthermore, multiparous women (≥3 live births) who never breastfed were at a higher risk of ER–/PR– breast cancers compared with multiparous women with a history of breastfeeding (57). A study by Harvell et al. analyzed the presence of breast cancer subtypes in PABC and found that the presence of Luminal A, Luminal B, Her2-positive, TNBC, and basal-like subtypes in PABC (58). Other studies also confirmed TNBC, Luminal B and HER2-positive as the most common subtypes among PABC while luminal A subtype was rare (25, 52, 59–61) (**Table 1**).

**Table 1:** Prevalence of molecular subtypes of breast cancer in pregnancy-associated breast cancer (PABC).

Population (Year)	Prevalence of Molecular Subtypes (%)				Reference
	Luminal A	Luminal B	HER2-Positive	TNBC	
Chinese (2020)	10.8%	30.4%	15.8%	17.4%	(61)
Chinese (2019)	7.1%	47.1%	22.9%	22.9%	(25)
Korean (2018)	7.7%–21%	21.1%	17.3%	35.9%–40.4%	(59, 60)
Hungarian (2014)	0%	32.8%	18%	48.4%	(52)

Several risk factors have been associated with PABC including hormonal changes, immune suppression during pregnancy as well as diagnostic challenges related to increased postpartum breast density and subsequent breast cancer diagnosis (14, 30). Breast involution is considered an important risk factor due to its shared features with pro-inflammatory microenvironment (26, 62–64), thus, providing suitable grounds for tumor growth and spread (65). Possible mechanisms of PABC include breast differentiation and involution (14, 66, 67). Following lactation, breast remodeling is a regulated program that involves the stimulation of fibroblasts, endothelial cells and immune cells. These cells then activate breast cells enhancing wound closure and remodeling of damaged tissue leading to the growth and development of transformed cells (14, 66). Moreover, an *in-vitro* and *in-vivo* study showed that involuting breast can assist the growth of existing tumor cells (66). *In-vivo* data showed that weaning-induced involution maintained ductal development of normal cells, however, in tumor cells they promoted invasion. Intriguingly, Yang et al. reported that early age at menarche, nulliparity, and late age at first birth increased the risk of luminal A breast cancer without any association with TNBC (68). On the contrary, all highlighted factors were identified as risk factor for TNBC in several other studies (69–74). Women’s race was also identified as a risk factor for TNBC. For instance, in comparison with white women, African-American women were found to be at a higher risk for TNBC, especially at a young age (<45 years) (71, 75, 76). A study by Ma et al. showed a protective effect of breastfeeding against development of TNBC (69). While data from the African American breast cancer epidemiology and Risk (AMBER) Consortium, showed that breastfeeding decreased the risk of TNBC associated with multiparity (77). Several other studies have revealed a significant correlation between PABC and high-grade breast cancers (16, 78–80); high grade morphology can be linked with PABC up to 10 years following pregnancy.

PABCs frequently display a higher incidence of the TNBC phenotype in comparison with cancers affecting nulliparous women. TNBCs comprise around 30%–40% of all PABC cases (52, 81) and are more likely to occur in recent pregnancy associated (within 1-2 years) breast cancers (52, 81). However, another study showed that TNBC risk can be present beyond 2 years postpartum; being one of the reasons for overall poor prognosis that characterizes tumors detected after pregnancy (16).

### Molecular Features of Pregnancy-Associated Breast Cancer

To further understand the carcinogenic molecular pathways effected during pregnancy, leading to breast cancer development, it is crucial to investigate associated gene deregulation patterns as well as mutations and their role in breast cancer development. In comparison with normal epithelium, in PABC several hormone target genes regulating the mitotic phase were overexpressed (58); four of these genes *MKI6, AURKA, BIRC5*, and *MMP11* are included in the Oncotype DX (82). Furthermore, the expression of tumor suppressor, p63 was downregulated in PABC; its expression correlates with enhanced invasion and aggressive feature of PABC (58, 83).

Azim et al. aimed at identifying the effects of pregnancy and involution on certain gene expression patterns in breast cancer cells compared with normal breast tissue (84). The authors found that the expression of *PD-1*, *PD-L1*, and gene sets related to *SRC*, *IGF1*, and *β-catenin* were higher compared with non-parous breast cancer females. However, this difference in the expression did not reach statistical significance. Therefore, in order to confirm this important finding with high statistical significance, more studies and larger patient sample sizes are necessary, which may lead to important therapeutic avenues based on these gene targets. During pregnancy, in response to growth hormones, expression of ER, PR, and IGF-1 is elevated and is linked with increase in breast cancer cell proliferation (14). In this context, *in-vivo* studies showed loss of *PD-L1* to correlate with fetal resorption and increase in fetal lethality (85, 86), hence the expression pattern of PD-1 and its ligand PD-L1 in PABC was assessed. Another study analyzed PD-1 and PD-L1 expression in both tumor-infiltrating lymphocytes (TILs) in PABC and nulliparous women; PD-L1 was strongly elevated in PABC TILs in comparison with controls, independent of tumor characteristics (87). On the other hand, in TILs PD-1 was expressed in both PABC and nulliparous women (87). Research has shown that high stromal TILs and PD-L1 expression to be frequently present in TNBC (88). A similar study by Acs et al. (2017) assessed PD-1, PD-L1, and CTLA-4 expression in PABC and non-PABC women; the expression of PD-1, PD-L1 was seen in peritumoral lymphocytes, however there was no expression of CTLA-4 and elevated PD-L1 expression was associated with early-onset of breast cancer and poor prognosis (89).

Furthermore, certain pathways were also found to be highly activated in parous breast cancer females including the G protein-coupled receptor (GPCR) and the serotonin receptor signaling pathways (84). GPCR signaling pathway plays a vital role in multiple cellular processes and mediates the activation of around 3% of the genes (90). Consequently, any aberration within this pathway may contribute to various diseases including cardiac, inflammatory and neoplastic (91). However, GPCRs are large family of receptors and only two of these receptors (CXCR4 and GPR30) were found to be highly expressed in breast cancers (84). The upregulations of these genes may induce breast cancer growth and metastasis (92). Moreover, activation of those GPCRs happens upon their binding to their ligands, which triggers the subsequent Ca2+ mobilization and kinase cascade activation leading to the induction of the expression of genes that are crucial for cellular growth (93).

The interplay between pregnancy and breast cancer has been an intriguing research topic over the years (94–96). Schedin et al. showed that many alterations- both inflammatory and non-inflammatory- occur in postpartum breasts causing a tumor inducing microenvironment (14). To further illustrate the mechanisms through which this takes place, Asztalos et al. reported that the involution process works to restore the status of breast tissue -prior to pregnancy- by inducing apoptosis, detachment of cells from the basement membrane (97), and other inflammation related events (62). The created inflammatory environment may initiate and contribute to the progression of breast cancer, especially through promoting tumor cell proliferation (14, 98). More importantly, a multitude of studies report different genetic patterns of PABC as compared to those detected in nulliparous women (16, 52, 81). Notably, estrogens can also bind to a known subtype of GPCRS, G protein estrogen receptor (GPER), thus contributing to breast cancer initiation and progression (99). The upregulation of serotonin induces tumorigenesis through induction of cellular proliferation (100). Furthermore, serotonin receptor pathway helps in the regulation of the expression of cathepsin S (CTSS) and is highly expressed in several cancer subtypes in which it correlates with their progression (101). The aggressiveness of pregnancy associated-TNBC, its poor prognosis and lack of treatment modalities makes it pivotal to study its genetic patterns and identify novel treatment options.

#### Molecular Features of Pregnancy Associated Triple-Negative Breast Cancers

Several variations are mainly ascribed to TNBC subgroup that was reportedly more predominant in PABCs (16, 52, 81); however, the exact mechanism by which pregnancy induces TNBC is yet to be fully elucidated. Among the different subtypes of breast cancer, CTSS is involved in invasion and is highly expressed in TNBC (102). Another factor that might contribute to the poor prognosis of TNBC is that TNBC increases the levels tryptophan-2,3-dioxygenase (TDO2) enzyme through inflammatory signals (103). TDO2 is a critical enzyme in catabolism of tryptophan, and its upregulation increases the production of tryptophan metabolites that exhibit antiapoptotic effects in TNBC cells (102).

Nevertheless, studies that specifically addressed the impact of pregnancy on development of TNBC are scarce. Asztalos et al. found a unique gene expression pattern for a specific set of genes in parous females who developed breast cancer in comparison to nulliparous breast cancer females. Differently expressed genes included 14 genes such as *CXCL1, CXCL12, ELN, ERBB2, ESR1, FBN1, IL1A, IL8, MMP12, MMP2, PGR, TGFB3, THBS1*, and *TIMP2*. Four of these genes (*CXCL1, IL1A, IL8, MMP12*) were upregulated, while the remaining 10 genes were down regulated in TNBC. Notably, downregulation of three of these genes (*ESR1, PGR, ERBB2*) are features of TNBC (104). Three of the upregulated genes (*CXCL1, IL1A, IL8*) are involved in inflammatory responses. Furthermore, inflammation and wound-healing involve macrophage cell influx, increased levels TGF-β1 and β3, MMPs-2, -3, and -9, and presence of fibronectin and laminin; these are linked with tumor progression and result in metastasis (14, 105–107). The interaction between cells and fibronectin *via* β_1_ integrins results in the onset of human breast cancer (108). Upregulated expression of TGF-β triggers matrix deposition and growth of fibroblasts in the healing wound, thus, accelerating tumor growth (107, 109). Indeed, MMPs are essential for the process of angiogenesis and lymphangiogenesis; both processes are essential in wound healing and tumor initiation and progression (110, 111). These findings further support the hypothesis that inflammation could contribute to the development of TNBC after pregnancy.

Interestingly, Azim et al. reported that the receptor activator of the nuclear factor κB ligand (RANKL) is found to be repressed in TNBC compared with other types of breast cancer, while the receptor activator for nuclear factor κB (RANK) was found to be highly expressed in TNBC (112). However, the link between these TNBC patients and pregnancy was not been found (113). **Table 2** summarizes function of the identified genes in normal cells and PABC.

**Table 2:** Genes reported to having unique pattern of expression in triple-negative breast cancers (TNBC) patients and their associated functions.

Gene	Function in Normal Cells	Function in PABC	Reference
*CTSS*	Promotes antigen processing.	Angiogenesis, tumor progression, and invasion.	(102)
*CXCL1*	Inflammation.	Decreased relapse-free survival and metastasis.	(114)
*CXCL12*	Embryogenesis, Inflammation, and Immunity.	Promotes tumor growth and metastasis.	(115)
*ELN*	Provides elasticity to organs and tissues.	Enhances tumor migration and progression.	(116)
*ERBB2/HER2*	Potentiates intracellular signaling.	Promotes metastasis and lower overall survival rates.	(117)
*ESR1*	Encodes estrogen receptor.	Downregulation is associated with worse outcome and poorly differentiated carcinomas.	(118)
*FBN1*	Structural support in elastic and non-elastic connective tissues.	Promotes tumor migration and invasion.	(119)
*IL1A*	Inflammation and hematopoiesis.	Promotes angiogenesis.	(120, 121)
*IL8*	Inflammation.	Promotes metastasis.	(122)
*MMP12*	Tissue remodeling.	Promotes angiogenesis and tumor progression.	(123)
*RANKL*	Regulation of T cell-dependent immune response.	Triggers endocrine therapy resistance and tumor progression.	(124)
*PD-1*	Negative regulator of immune response.	Promotes cancer immune evasion.	(125)
*Src*	Involved in embryonic development and cell growth.	Promotes malignancy and is associated with poor prognosis.	(126)
*TGF-β3*	Aids in embryogenesis, cellular differentiation and wound healing.	Promotes tumor progression and is associated with poor prognosis.	(127)
*THBS1*	Mediates cell-to-cell and cell-to-matrix interactions.	Involved in platelet aggregation, angiogenesis, and tumorigenesis.	(128)
*TIMP2*	Suppress proliferation of endothelial cells and maintain tissue homeostasis.	Promotes progression of cancer.	(129)

All information regarding the functions of those genes was collected form GeneBank (https://www.ncbi.nlm.nih.gov/genbank/).

Tumor suppressors, *BRCA1/2* are involved in DNA damage repair, cell cycle control, transcription and ER type alpha activity (130). Mutations in *BRCA1/2* are considered as risk factors for the onset of breast cancer (131); Atchley et al. reported a significant association between mutations in breast cancer susceptibility gene 1 (*BRCA1*) and TNBC, with more than 2/3 of *BRCA1* mutations cases being of TNBC phenotype (132). Earlier studies have indicated that *BRCA1/2* mutation carriers can be at a higher risk for PABC (133, 134). A study by Johannsson et al. analyzed the incidence of PABC in carriers of *BRCA1* and *BRCA2* mutations in comparison with premenopausal Swedish women aged ≤ 40 with sporadic PABC (133). The study showed that *BRCA1/2* carriers are at an increased risk for PABC and hence should be monitored carefully during pregnancy and in the postpartum period (133). Another study revealed a significantly higher (25%) PABC frequency among *BRCA1/2* mutation carriers compared with non-PABC cases (135). Although deleterious *BRCA1* mutations are frequently encountered on both sporadic and hereditary TNBC (136), no link between pregnancy and these mutations has been found. Similarly, no association between TNBC and mutations in *BRCA2* gene were reported (137). Based on the previous discussion, we underline here that the exact link between pregnancy and TNBC remains to be elucidated.

#### Signaling Networks in Pregnancy-Associated Breast Cancer

**Figure 1:**
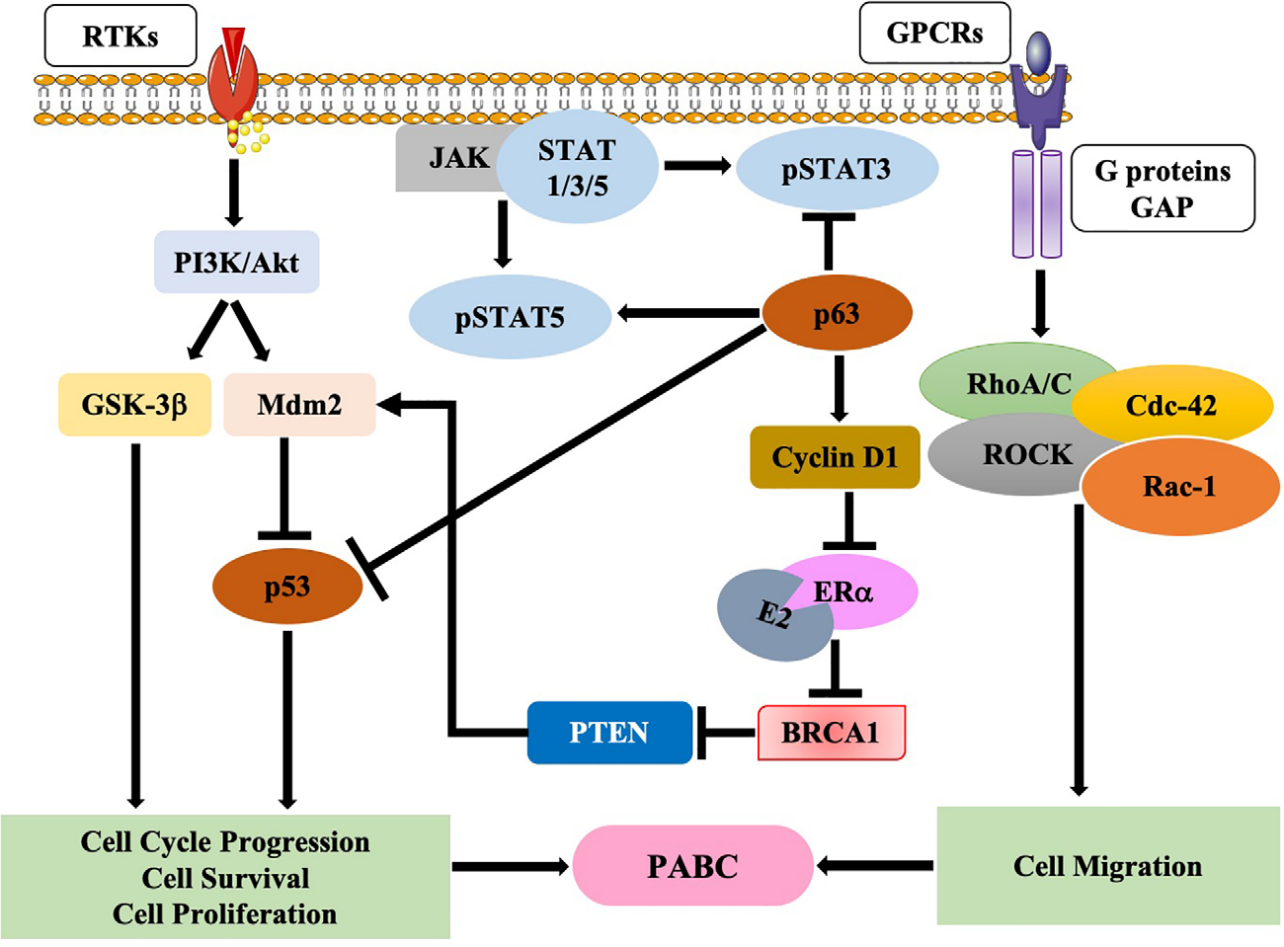
Molecular pathways in pregnancy-associated breast cancer (PABC). Schematic diagram showing various pathways that are involved in the onset and progression of PABC.

Earlier investigations suggested various underlying molecular mechanisms underpinning the onset of PABC. In an *in-vivo* study by Wagner et al. (138), using WAP-Cre/Rosa-LacZ transgenic mice, the authors identified a mixed population of alveolar cells called parity-induced mammary epithelial cells (PI-MECs) in the mammary gland of parous, non-pregnant female mice; these cells were not present in nulliparous females. PI-MECs rely on the transcription factor p63 for survival (138–142); one-time pregnant mice (MMTV–Her2/Neu mouse model) lacking p63 have lower tumors, thus indicating a tumor-promoter role for PI-MECs (138). Furthermore, another study showed that increased expression of p63 inhibits the p53 and STAT3 pathways; however, p63 enhances the expression of the pro-survival signaling STAT5 pathway, thus, initiating PI-MEC-induced tumorigenesis (142) (**Figure 1**). On the other hand, another study found loss of p63 during pregnancy reduced cyclin D1 levels, thus, suggesting a role of p63 in inducing PI-MEC survival post-partum (143). As shown in **Figure 1**, p63 levels increases cyclin D1which in turn inhibits estrogen receptor-alpha (ER-a) transactivation, further inhibiting the expression of BRCA1 (144). Following BRCA1 inhibition, PTEN is inactivated thereby activating mdm2 and blocking p53 (145) (**Figure 1**), which leads to genomic instability. Alternatively, pregnancy aids premalignant MECs evasion of apoptotic signaling through the activation of the JAK-STAT5 axis (146–148). In addition, receptor tyrosine kinases initiate downstream oncogenic signaling pathways such as PI3K/Akt which further activate either glycogen synthase kinase 3-beta (GSK3-β) or mdm2 (**Figure 1**).

On the other hand, in pregnant women, GPCRs are activated (84), these growth factors enhance focal adhesion kinase (FAK) which in turn activates RhoA/Rac1/cdc42/ROCK complex, thereby initiating cell migration (149) (**Figure 1**).

However, further understanding of the underlying molecular mechanisms of PABC is needed to help pave the way for the development of possible therapeutic strategies.

### Diagnosis of Pregnancy-Associated Breast Cancer

Since pregnancy associated TNBCs have a poor prognosis and diagnostic delays may occur in pregnancy due to effects of pregnancy related hormones, increased awareness can help in paving the way for appropriate treatment.

Vis a vis PABC diagnosis, assessing breast symptoms during pregnancy and postpartum period can be perplexing due to hormonally induced changes in breast tissue that can result in augmented firmness and nodularity (150). Furthermore, postpartum lactational mastitis symptoms may mimic locally advanced or inflammatory breast cancer. The majority of PABCs are diagnosed after presenting with a palpable mass (151). To determine the scope of disease is critical in treatment decision-making.

Breast imaging includes mammography, ultrasound, and magnetic resonance imaging (MRI). While ultrasound is proven to be safe and commonly used in pregnancy (152), mammography confers minimal dose to the fetus with abdominal shielding (153). Contrast-enhanced breast MRI can be a useful diagnostic tool in non-PABC, however, in pregnancy, the safety of gadolinium still remains controversial. A free gadolinium is considered toxic as it can cross the placenta and stay in the amniotic fluid, which can be taken in by the fetus re-entering the fetal circulation (154). In addition, prone positioning required during breast MRI can apply a sustained pressure on the gravid uterus, disrupting uterine blood flow (154). In case of metastatic PABC, diagnostic workup prior to the delivery is required to enable therapeutic interventions. Generally, the pregnant patient is subjected to either chest X-ray with abdominal shielding, liver ultrasound or non-contrast supine MRI to check for lung, liver or bone metastasis, respectively (153).

Based on the imaging results, biopsy [fine needle aspiration (FNAC) or core needle biopsy (CNB)] is done for definite diagnosis of a breast mass (155). Although, FNAC is less traumatic with a low complication rate than CNB and generally does not require local anesthesia, FNAC provides inadequate information about the histopathological type, grade, steroid receptors, HER2 expression, and intrinsic behavior of the tumor (155, 156). Hence, CNB is considered as a more reliable method of pathological diagnosis of breast cancer (156). The tissue obtained from a biopsy is tested to determine the status of the hormone receptors (ER, PR) as well as Her2 and proliferation index (Ki-67) (157). Biopsies are performed either under ultrasound or stereotactic guidance (158). In addition, during the first and second trimesters of pregnancy, incisional or excisional biopsy can be safely done (159).

Once diagnosis of breast cancer has been completed by imaging methods and histopathology, it is essential not to postpone the treatment. It can be given post-delivery if the patient is in near term. If the patient is close to term, the treatment must commence (160).

### Treatment of Pregnancy-Associated Breast Cancer

Treatment options for PABC remain challenging and may require special considerations. Surgery (e.g., modified radical mastectomy) is usually considered as the primary line of treatment in breast cancer during pregnancy but neoadjuvant chemotherapy has been widely used as a primary treatment option for advanced HER2-positive and TNBC (161). There are several concerns regarding the use of neoadjuvant chemotherapy that pertain to the potential peripartum complications and the impact on the fetal outcome (162). However, studies have shown that during the first trimester, chemotherapeutic agents are not advised as they may be potentially teratogenic (161). On the other hand, after completion of the first trimester, chemotherapeutic agents may be safely administered without the risk of fetal malformations (161). Multiple studies that explored the use of chemotherapeutic drugs for the treatment of breast cancer during pregnancy showed that the majority of drugs (taxanes and vinorelbine) are non-toxic during the second and third trimesters of pregnancy. However, these drugs may increase the risk of intrauterine growth restriction and preterm labor. Cytotoxic drugs may also induce both maternal and infant leukopenia, hence, chemotherapy after 35 weeks of gestation is contraindicated to avoid delivery of a leukopenic infant (161). Other drugs including methotrexate, trastuzumab and tamoxifen should also be avoided during pregnancy due to their effect on the central nervous system, cardiac, gastrointestinal and skeletal malformations, oligohydramnios (low levels of amniotic fluid), preterm labor, and spontaneous abortions (163–165). All these considerations should be taken into account when optimizing treatment options of breast cancer during pregnancy. A recent 4^th^ ESO-ESMO guideline also emphasizes the need of an individual basis approach following the international guidelines and an expanded multidisciplinary team that will involve gynecologists/obstetricians as well as perinatologists, in addition to patients’ own preferences (166).

Adjuvant chemotherapy is helpful and encouraged in patients with high-risk breast cancer including those with PABC. High-risk prognostic factors include estrogen and progesterone receptor negative status, HER2 status, high tumor grade, high TNM, and younger age of the patient (167). Patients that are treated with neoadjuvant chemotherapy and extensive residual disease (burden) are also strong candidates for adjuvant chemotherapy.

Radiation therapy is not advised during pregnancy as it can pose a high risk for fetal toxicity and malformations, childhood cancers and delays in neurocognitive development (168, 169). However, adjuvant radiotherapy (postpartum) can be safely used as in other breast cancer cases and following strict indications for adjuvant radiotherapy.

Breast cancer in young women (age < 40 years) tend to recur and therefore younger age of diagnosis, and hence longer lifespan places these patients at a statistically increased risk of recurrence and distant metastasis over time (170). Van Nes and van de Velde recommended mastectomy in younger patients over breast-conserving treatment (170). Any delay in treatment due to fallacies regarding risk of local and systemic therapy may worsens oncologic outcomes. Ambiguities regarding the safety of diagnostic modalities and treatment of PABC may lead to worse outcomes in the group of younger pregnant women with breast cancer. However, recent studies provide robust data on the safety of diagnostic procedures that can enable a successful treatment of patients with this challenging malignancy.

Sentinel lymph node biopsy (SLNB) is a part of routine management of breast cancer and has been widely used in clinical practice. SLNB recommendation is proposed for patients with clinically node negative breast cancers, those with or without 1–2 suspicious lymph nodes on imaging, and for patients that were not treated with neoadjuvant systemic therapy (171). In contrast to the American Society of Clinical Oncology (ASCO) guidelines, the National Comprehensive Cancer Network (NCCN) guideline indicates lack of scientific evidence regarding the use of SLNB in pregnant women. While, the NCCN also advises that SLNB use should be an individualized decision, but not directly offered to pregnant women < 30 weeks’ gestation. Of note, NCCN does not recommend the use of isosulfan or methylene blue dyes for SLNB in pregnancy while use of radioactive tracer (e.g., technetium 99m sulfur colloid) is also supported by limited scientific data regarding the fetal radiation dose (171).

## Conclusions And Future Directions

PABC incidence increases as women choose delayed childbirth, and while it is a rare form of breast cancer with a significant propensity for triple-negative phenotype; Nevertheless, PABC is a diagnostically and therapeutically challenging disease bearing various risks for affected woman and fetus. Although immunotherapy with immune checkpoint inhibitors may be a promising therapeutic approach for patients with PABC, it can trigger various autoimmune side effects or immune-related adverse events (irAEs); there are several endocrine-related irAEs (172). The most common endocrinopathies reported from clinical trials include hypothyroidism and hypophysitis in patients treated with anti-PD-1/PD-L1 antibodies and anti-CTLA4, respectively (173–178). In addition, hypopituitarism, type 1 diabetes mellitus and primary adrenal insufficiency have also been reported (172). On the other hand, treatment with Dasatinib alone or combined with immune checkpoint inhibitors could be another therapeutic rationale given that PABC frequently overexpress the corresponding receptors Src and PD-L1. Nevertheless, despite the overall poor outcome, we believe that the complete gene and miRNA profiles of PABC can aid in identifying novel therapeutic targets and biomarkers to manage this rare, but fatal disease. In conclusion, the etiology of PABC remains largely unknown, thus, further cellular and animal models in addition to preclinical and clinical studies in the field are necessary.

## Author Contributions

AA conceptualized the study. SA, IS, SM, HFA, SV and AA wrote, reviewed, and edited the manuscript. AA, HFA and SV acquired the funding. All authors have read and agreed to the published version of the manuscript. All authors contributed to the article and approved the submitted version.

## Funding

Our lab is supported by grants from Qatar University: # QUCP-CMED-2019-1, QUHI-CMED-19/20-1, and QUCG-CMED-20/21-2.

## Conflict of Interest

The authors declare that the research was conducted in the absence of any commercial or financial relationships that could be construed as a potential conflict of interest.

## Abbreviations

AMBER, African American breast cancer epidemiology and Risk Consortium; ASCO, American Society of Clinical Oncology; AURKA, Aurora Kinase A; BIRC5, Baculoviral IAP Repeat Containing 5; BRCA, Breast cancer susceptibility gene; Ca2+, Calcium ions; cdc, Cell division control protein; CNB, Core needle biopsy; CTLA4, Cytotoxic T-Lymphocyte Associated Protein 4; CXCL, C-X-C Motif Chemokine Ligand; CXCR, C-X-C Motif Chemokine Receptor 4; ELN, Elastin; ER/ESR, Estrogen receptor; ERBB2, Erb-B2 Receptor Tyrosine Kinase 2; FAK, Focal adhesion kinase; FBN, Fibrillin; FNAC, Fine needle aspiration; GPCR, G protein-coupled receptor; GSK3-β, glycogen synthase kinase 3-beta; HER2, human epidermal growth factor receptor 2; IGF1, Insulin Like Growth Factor 1; IL, Interleukin; ILC, Invasive lobular carcinoma; irAEs, Immune-related adverse events; JAK, Janus Kinase; LAR, Luminal androgen receptor; mdm2, Mouse double minute 2 homolog; MMP, Matrix metallopeptidase; MMTV, Mouse mammary tumor virus; MRI, Magnetic resonance imaging; NCCN, National Comprehensive Cancer Network; NST, No-special-type; p53, Tumor protein 53; p63, Tumor protein 63; PABC, Pregnancy-associated breast cancer; PD-1, Programmed cell death protein 1; PD-L1, Programmed cell death-ligand 1; PGR/PR, Progesterone receptor; PI-MECs, Parity-induced mammary epithelial cells; PTEN, Phosphatase And Tensin Homolog; Rac1, Rac Family Small GTPase 1; RANKL, Receptor activator of the nuclear factor κB ligand; RhoA, Ras Homolog Family Member A; ROCK, Rho Associated Coiled-Coil Containing Protein Kinase; SLNB, Sentinel lymph node biopsy; src, SRC Proto-Oncogene, Non-Receptor Tyrosine Kinase; STAT, Signal Transducer And Activator Of Transcription; TDO2, Tryptophan-2,3-dioxygenase; TGF, Transforming growth factor; TILs, Tumor-infiltrating lymphocytes; TNBC, Triple-negative breast cancer.
